# Injectable and In Situ Gelling Dextran Derivatives Containing Hydrolyzable Groups for the Delivery of Large Molecules

**DOI:** 10.3390/gels7040150

**Published:** 2021-09-24

**Authors:** Laura Di Muzio, Patrizia Paolicelli, Chiara Brandelli, Stefania Cesa, Jordan Trilli, Stefania Petralito, Maria Antonietta Casadei

**Affiliations:** Department of Drug Chemistry and Technologies, Sapienza University of Rome, 00185 Rome, Italy; laura.dimuzio@uniroma1.it (L.D.M.); chiara.brandelli@uniroma1.it (C.B.); stefania.cesa@uniroma1.it (S.C.); jordan.trilli@uniroma1.it (J.T.); stefania.petralito@uniroma1.it (S.P.); mariaantonietta.casadei@uniroma1.it (M.A.C.)

**Keywords:** dextran derivatives, PEG methacrylate, photo-crosslinking reaction, biodegradable hydrogels, injectable hydrogels, in situ gelling polymers, macromolecules delivery

## Abstract

Recently, we reported the synthesis and characterization of a new dextran derivative obtained by grafting polyethylene glycol methacrylate to a polysaccharide backbone through a carbonate bond. This moiety was introduced because it allows for the fabrication, through a photo-induced crosslinking reaction, of biodegradable hydrogels particularly suitable for the release of high molecular weight molecules. Here, we investigate the influence of the oxyethylene chain length and the molecular weight of the starting dextran on the main properties of the polymeric solutions as well as those of the corresponding hydrogels. All synthesized polymeric derivatives were characterized by FTIR, NMR, and rheological analyses. The photo-crosslinking reaction of the polymers allowed us to obtain biodegradable networks tested for their mechanical properties, swelling, and degradation behavior. The results showed that both the oxyethylene chain length as well as the molecular weight of the starting dextran influenced swelling and degradation of the hydrogel network. As a consequence, the different behaviors in terms of swelling and degradability were able to affect the release of a large model molecule over time, making these matrices suitable candidates for the delivery of high molecular weight drug substances.

## 1. Introduction

Hydrogels are, in general, considered biocompatible materials due to their high water content and their consistency, which make them similar to the extracellular matrix, minimizing tissue irritation and inflammatory response when used for biomedical or pharmaceutical purposes [1,2,3,4,5]. The porous structure of these networks can house large quantities of water-soluble molecules, so the interest in the use of hydrogels as drug delivery systems is constantly growing [6,7]. The release of active molecules physically incorporated into the hydrogel matrix can be governed by multiple mechanisms such as swelling, diffusion, erosion/degradation, or by the combination of two or more of these mechanisms [8,9]. In particular, if the hydrogel mesh size is larger than the drug hydrodynamic radius, then the diffusion is the driving force for the release process. If, instead, the pores are smaller than the radius of the loaded molecule, swelling and/or degradation processes are required to allow for drug release [10,11,12]. Considering the internal structure of hydrogels, the release profile can be modulated by varying the lattice density, and, therefore, the polymer architecture, the molecular weight, the concentration, or the chemistry of the system [3].

For the purpose of drug delivery, biodegradable hydrogels are the platform of choice, particularly for the delivery of high molecular weight drugs [13]. Indeed, a successful strategy that allows the controlled release of large molecules is to design a system so that biodegradability and bioerodability can be modulated and opportunely tailored. Hydrogel degradation not only allows for temporal control of the release of the entrapped bioactive molecule, but it also makes possible the elimination of the carrier once the payload is completely depleted. Hydrogel degradation can occur in the polymer backbone or at the crosslinking points, mediated either by enzymatic or chemical processes, and can be controlled by studying the progressive erosion and rupture of the polymeric network. As a consequence, biodegradable materials can break down inside the body, producing water-soluble intermediates or end-products that can be easily eliminated from the body without harmful or toxic effects.

Based on their degradable features, hydrogels can deliver the drugs following predictable release profiles, at the desired site of action, with no burst effect. All these features make biodegradable hydrogels a really good platform for drug delivery. When used as preformed systems, they must be surgically implanted in the site of interest, but this operation is usually expensive and disadvantageous for the patient. For this reason, nowadays, the interest has shifted to the design of hydrogels that can be administered in less invasive ways such as injectable hydrogels [14,15]. Indeed, injectable hydrogels can be administered as polymeric solutions that convert to viscoelastic systems at the injection site [16,17]. Gelation can occur as a result of external stimuli (such as temperature, pH, and ionic strength) or through the use of physical or chemical tools. In particular, the use of methacrylated polymers allows hydrogels to be produced by UV irradiation. In this scenario, dextran (DEX) functionalized with methacrylic moieties has proven to be an interesting platform for drug delivery [18]. This meanly occurs when methacrylic groups are coupled to a DEX backbone with hydrolyzable carbonate ester bonds, giving the so-called dextran hydroxyethyl methacrylate (DEX-HEMA). While DEX-HEMA forms hydrogels with improved degradability compared to classic dextran methacrylate (DEX-MA—methacrylic groups directly linked to the polysaccharide through ester bonds), the release and rheological properties are still suboptimal for effective application as an injectable and in situ crosslinkable drug delivery system. Therefore, we recently proposed a new derivative coupling DEX with polyethylene glycol methacrylate (PEG-MA) through a carbonate ester bond [19]. The presence of PEG was able to positively affect the mechanical properties of the hydrogels, while the carbonate group allowed for the biodegradation of the network as well as temporal control of the release of an incorporated drug, particularly a high molecular weight drug. Here, we investigate the influence of the length of the oxyethylene lateral chain as well as the molecular weight of DEX on the swelling, mechanical, and release properties of the obtained hydrogels to further control and modulate their biodegradation. To this end, five DEX derivatives were synthesized, fully characterized, and employed for the preparation of hydrogels by photo-induced free radical crosslinking. A large model molecule was loaded into the hydrogels and its release was followed over time.

## 2. Results and Discussion

### 2.1. Synthesis of DEX Derivatives

A series of DEX derivatives was synthesized with the aim to introduce methacrylic moieties on the polysaccharide backbone through labile carbonate ester groups, in order to modulate biodegradability of the corresponding photo-crosslinked hydrogels, and in this way obtain suitable and tailorable carriers for the delivery of high molecular weight drugs.

**Figure 1:**
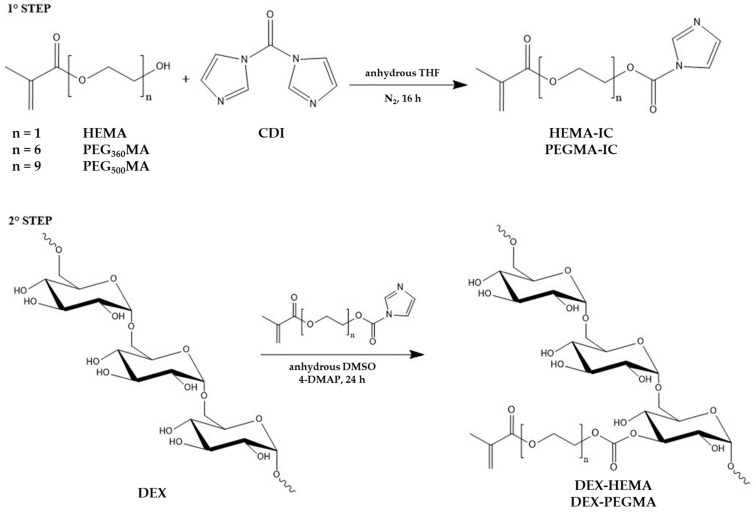
Scheme of the synthesis of methacrylated DEX derivatives.

Spacers of different lengths, containing a different number of oxyethylene units, namely one, six, or nine, were employed to link methacrylic groups to the polymer backbone. To this end, the following methacrylate reagents were used: HEMA, PEG_360_MA, and PEG_500_MA. Moreover, as the final goal of the work was the development of an injectable and in situ gelling delivery system, the effect of the molecular weight of the starting DEX was also evaluated. Therefore, both DEX_40_ (M_r_ 40,000) and DEX_500_ (M_r_ 500,000)-based methacrylate derivatives were synthesized. The chemical modification of DEX was obtained following a two-step synthesis procedure, as shown in the scheme reported in Figure 1. In the first reaction the hydroxyl group of the methacrylic reagent was activated by the classic reaction with *N*,*N*’-carbonyldiimidazole (CDI) to produce a reactive methacrylic reagent. After evaporation of the solvent, the intermediate was directly put to react, without prior purification, with DEX in anhydrous DMSO, using 4-DMAP as a nucleophilic catalyst.

The same procedure was followed to synthesize all the derivatives, but the polymers obtained from DEX_500_ were precipitated from the reaction mixture using 2-methoxyethanol instead of ethanol, because it allowed for the formation of a fine precipitate, which dissolved rapidly in distilled water, whereas ethanol produced a dense precipitate, which dissolved slowly in water, thereby promoting partial degradation of the polymer. Anyway, the derivative with the highest molecular weight (DEX_500_-PEG_500_MA) showed poor water solubility even using 2-methoxyethanol for purification, and for this reason, it was discarded and not further considered in this work.

**Figure 2:**
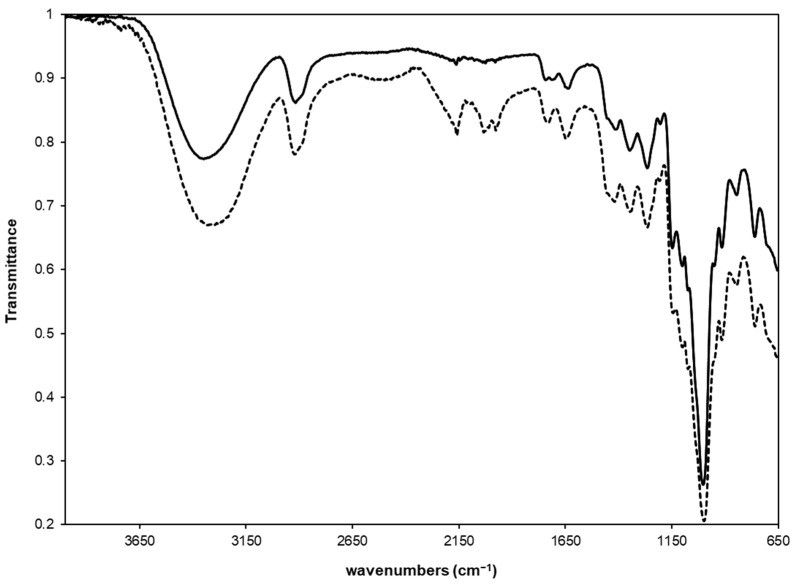
FTIR spectra of DEX_40_-PEG_360_MA (solid line) and the corresponding hydrogel (dashed line). The insert represents an expanded region of the spectra from 1900 to 1500 cm^−1^.

After exhaustive dialysis and freeze-drying, every derivative was submitted to FTIR analysis. As an example, in Figure 2, the spectrum of DEX_40_-PEG_360_MA is reported, however, similar results were obtained for all other polymers.

As a consequence of the derivatization, the FTIR spectrum showed the presence of a peak at 1745 cm^−1^ due to the stretching of the carbonate ester bond and another one at 1713 cm^−1^, characteristic of the carbonyl group of the methacrylic ester.

**Figure 3:**
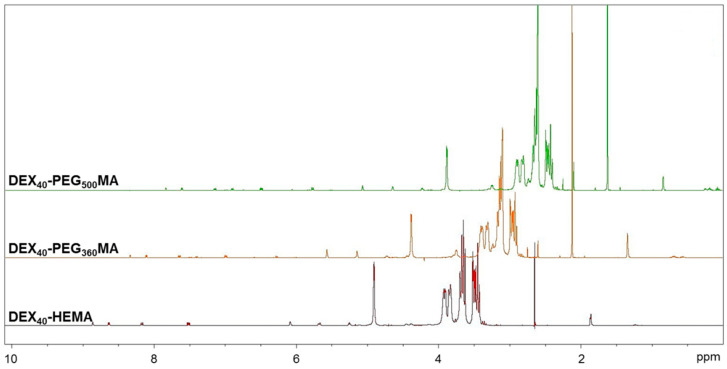
^1^H-NMR spectra of DEX_40_ derivatives in D_2_O/DMSO-d6.

The polymers were also characterized by ^1^H- and ^13^C-NMR. The ^1^H-NMR spectra reported in Figure 3 confirmed the functionalization. In fact, it was possible to identify the characteristic signals of the vinyl protons at 6.09 and 5.67 ppm and the methyl protons at 1.87 ppm of the methacrylic group [20].

^1^H-NMR spectra were also used to calculate the methacrylation degree (DD%) of the polymers. For this achievement, the ^1^H-NMR spectra were recorded in the presence of an internal standard. Nicotinamide was chosen as the internal standard, as the peaks related to the aromatic protons were in areas of the spectrum where no proton signals of the polymers under examination were present. The internal standard method was used since the methods usually used to calculate the methacrylation degree of DEX derivatives has been proven not to be accurate [21]. One of the most widely applied method calculates the DD% as the ratio between the average of the proton areas of the methacrylic groups (6.09 and 5.67 ppm) and the anomeric proton area (4.69 ppm). However, the resulting DD% values were poorly reproducible and scarcely accurate due to the presence of the water signal, which falls very close to the anomeric proton, preventing its accurate integration. Moreover, the suppression of the water signal partially alters the peak intensity of the anomeric proton, causing an overestimation of the DD% value. The other method usually used to calculate the methacrylation degree considers the signals of the DEX chain in the range 3.2–4 ppm. However, it was not possible to use this method because the signals of the methylene protons of the oxyethylene chain also fall within this range. For all these reasons, the internal standard method was selected and applied. By comparing the area of a specific standard signal with that relating to the protons of the methacrylic groups and precisely knowing the amount of standard in the sample, it was possible to accurately calculate the mmoles of the methacrylic groups of all the polymeric derivatives. The experimental conditions of the synthesis were opportunely optimized to obtain all the derivatives with DD% = 5 ± 1. This specific value was considered an ideal balance between the hydrophilic and lipophilic portion of the polymer, according to previous results [19].

**Figure 4:**
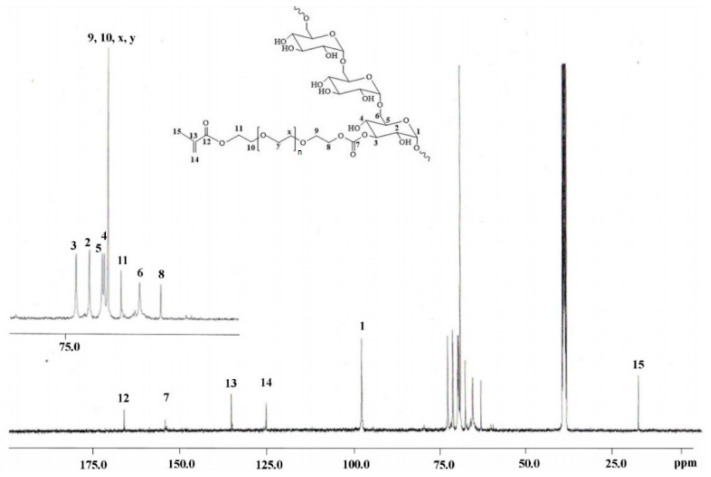
^13^C-NMR spectrum of DEX_40_-PEG_360_MA in DMSO-d6.

^13^C-NMR spectra of the derivatives show peaks that can be referred to the methacrylic group and the oxyethylene chain. As an example, Figure 4 reports the ^13^C-NMR spectrum of DEX_40_-PEG_360_MA. The signal at 167.0 ppm belongs to the carbonyl ester, whereas those at 136.3 and 126.3 ppm were the resonance peaks of the carbons of the methacrylic double bond; instead, the peak of the methyl group could be observed at 18 ppm. In addition, at 70.3, 66.6, and 64.2 ppm, it was possible to identify the chemical shifts of the carbons of the oxyethylene chain. Finally, the peak at 155.2 ppm can be referred to the carbonate ester group. Similar results were obtained for the other derivatives.

### 2.2. Rheological Characterization of Polymeric Solutions

**Figure 5:**
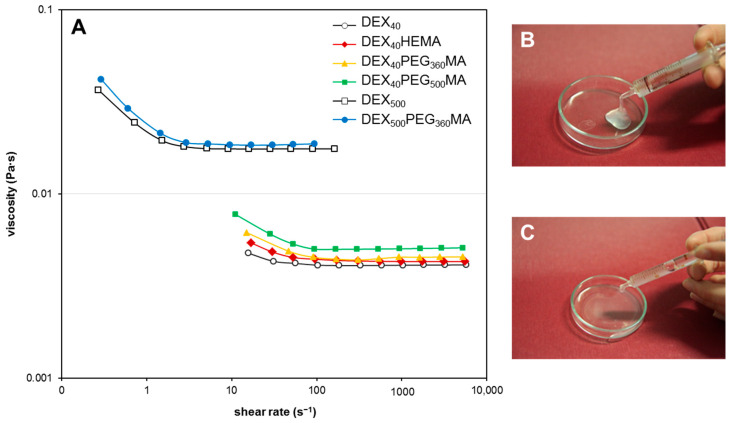
Flow properties of the different DEX derivatives. (**A**) Flow curves of aqueous solutions (10% *w*/*v*) of the derivatives at 37.0 ± 0.1 °C, showing the effect of the oxyethylene chain length and the molecular weight of DEX on the flowing properties of the polymers. (**B**,**C**) Show injectability of DEX_500_-PEG_360_MA and DEX_40_-PEG_360_MA, respectively.

In order to evaluate the possible use of these DEX derivatives as injectable drug delivery systems, their 10% *w*/*v* aqueous solutions were subjected to rheological analysis of the flow properties. The solutions displayed the behavior shown in Figure 5A. All systems showed the same decreasing trend of viscosity as a function of the flow rate, confirming the pseudo-plastic behavior typical of polymeric solutions. The slight increase in viscosity observed for the solutions of DEX-PEGMA polymers with respect to DEX-HEMA could be due to the formation of clusters among PEG chains [22,23] where DEX-HEMA is unable to form. Such interactions increase with the length of the PEG chain. As expected, when the molecular weight of DEX increases, the viscosity increases significantly. In fact, the viscosity of DEX_500_ and its PEGMA derivatives was significantly higher than DEX_40_ and its PEGMA derivatives, at the same concentration. Even in this case, the small difference in viscosity between the two derivatives can be related to cluster formation among the oxyethylene chains. However, the zero-shear viscosity of DEX_500_-PEG_360_MA is adequate for its use as an injectable system, and is also high enough to avoid the rapid spread of the polymeric solution in the injection site (see Figure 5B,C).

### 2.3. Hydrogels Preparation and Mechanical Characterization

Hydrogels were prepared by photo-induced free radical crosslinking of 10% *w*/*v* solutions of the different DEX derivatives. An irradiation time of 10 min was found optimal for the conversion of all the solutions into the corresponding hydrogel systems. Once obtained, gels were freeze-dried and analyzed through FTIR to confirm the achievement of the polymer crosslinking in all the matrices. In fact, in the FTIR spectra of the gels, the peak relative to the stretching of the methacrylic ester at 1713 cm^−1^ disappeared, while that of the saturated ester that formed after the photochemical reaction shifted toward the stretching vibrational band of the carbonate ester bond (see the dashed line in Figure 2).

**Figure 6:**
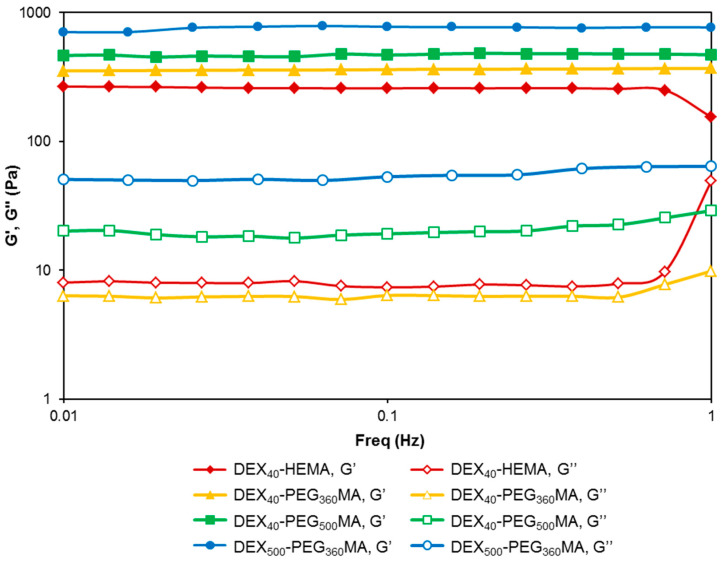
Frequency sweep analysis of all the methacrylated DEX derivatives showing the effect of the oxyethylene chain length and the molecular weight of the polymer backbone on the mechanical properties of the hydrogels (G’ closed symbols, G’’ open symbols). The spectra were recorded at 37.0 ± 0.1 °C in the linear viscoelastic region.

Once prepared, the hydrogels were characterized for their mechanical properties. The study of the mechanical properties is crucial to evaluate the handling and elasticity of the hydrogels. The samples were subjected to rheological analysis under small amplitude oscillatory shear conditions, and the relative mechanical spectra within the linear viscoelastic regime of the materials were recorded (Figure 6).

All samples showed the typical behavior of gels, with the elastic modulus G’ greater than the viscous modulus G’’. It can be seen that all the derivatives obtained from DEX_40_ showed an almost similar behavior with regard to the trend of the G’ and G’’ moduli. Furthermore, the absolute values of the two moduli depend on the oxyethylene chain length and increase with it. Considering that the mmoles of crosslinkable methacrylic groups slightly decrease in the order DEX_40_-PEG_500_MA < DEX_40_-PEG_360_MA < DEX_40_-HEMA, these results suggest that longer oxyethylene chains promote interactions between polymer chains. Therefore, moving the methacrylic groups away from the polymer backbone facilitates the successive crosslinking and formation of the network. The hydrogel obtained from DEX_500_-PEG_360_MA showed the highest values of the two moduli. Therefore, comparing DEX_40_-PEG_360_MA and DEX_500_-PEG_360_MA, the strength of the gel increased with the molecular weight of the starting DEX. This result is in accordance with the viscosity values obtained for the corresponding polymeric solutions and could reflect the greater extent of polymer chain entanglements characteristic of the derivative with higher molecular weight.

**Figure 7:**
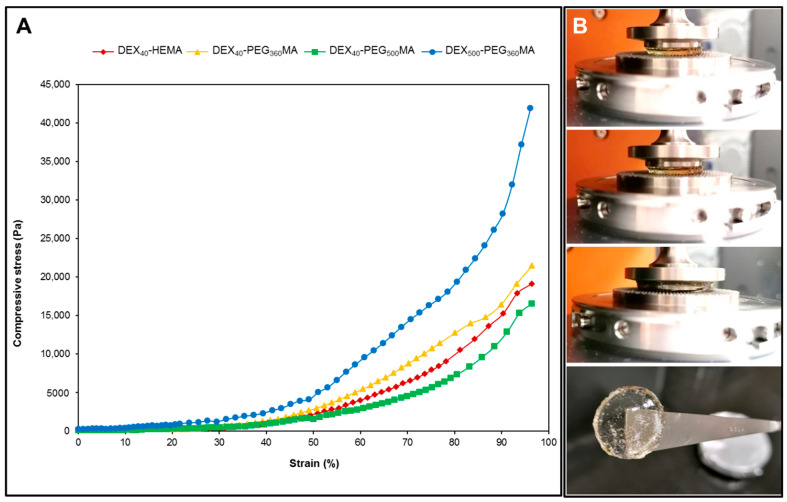
Dynamic mechanical analysis of the hydrogels in compression mode. (**A**) Stress–strain curves of all the methacrylated DEX derivatives submitted to uniaxial compression. (**B**) Sequence of images of DEX_40_-PEG_500_MA hydrogel submitted to uniaxial compression and the same hydrogel recovered at the end of the compression test.

Stress–strain profiles were obtained by subjecting the samples to uniaxial compression and the results are reported in Figure 7.

The slope of the curve in the range from 0 to 10% of strain was used to calculate the values of the compressive modulus. The length of the oxyethylene chain did not significantly influence the modulus value of the gels when submitted to uniaxial compression. Additionally, it can be observed that the DEX_40_-PEG_500_MA derivative showed a different behavior compared to DEX_40_-HEMA and DEX_40_-PEG_360_MA as it can be deformed easier than the other hydrogels, which were obtained from polymers with shorter lateral chains. Indeed, it required the application of lower stress for its compression. In contrast, the increment of the molecular weight of DEX contributed to producing a stiffer structure that requires much higher stress for deformation, probably due to the higher degree of polymer chain entanglement. All the hydrogels were crushed to the maximum allowed by the instrument; moreover, no samples broke during the test, as can be observed in Figure 7B, which shows a sequence of images of the DEX_40_-PEG_500_MA hydrogel submitted to uniaxial compression and the same hydrogel recovered at the end of the compression test. Similar results were obtained for all the hydrogels analyzed.

Overall, it is possible to attest that the mechanical properties of the hydrogels were mostly influenced by the molecular weight of the starting DEX, and to a smaller extent by the length of the oxyethylene chain used to link the methacrylate group to the polymeric backbone.

### 2.4. Swelling and Degradability Properties

**Figure 8:**
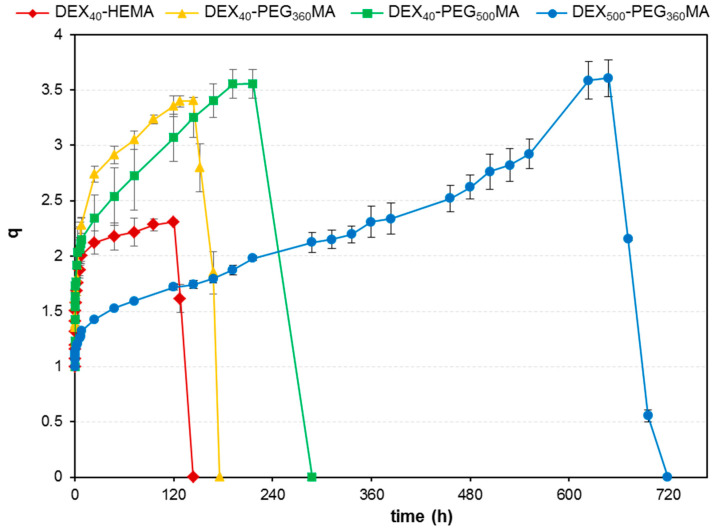
Dynamic swelling profile of all the methacrylated DEX derivatives measured in PBS (pH 7.4) at 37.0 ± 0.5 °C.

Apart from the mechanical properties, it is important to investigate the swelling and degradability properties of the different matrices, as they can deeply influence their performance as drug delivery systems, and, specifically, the amount of drug released over time. Swelling experiments were carried out on freeze-dried gels in PBS (pH 7.4) at 37.0 ± 0.5 °C. The results are reported in Figure 8 as value of q = W_s_/W_d_, where W_s_ and W_d_ are the weight of the swollen and dry sample, respectively. Figure 8 shows that the time needed to reach the maximum swelling degree is very different for the various networks, demonstrating how much the length of the spacer as well as the molecular weight of the backbone can affect the physical–chemical properties of these systems. Specifically, it should be noted that DEX_40_-HEMA-based hydrogel reached the maximum q value after 120 h, while those based on DEX_40_-PEG_360_MA, DEX_40_-PEG_500_MA, and DEX_500_-PEG_360_MA after 144, 216, and 648 h, respectively. As the molecular weight of DEX and the spacer length increase, the swelling rate slows down. Another peculiarity of these systems is that the maximum swelling degree is not very different, albeit reached in different times. However, DEX_40_-HEMA-based hydrogels are an exception to this behavior, probably due to its faster degradation. It was interesting to compare the swelling of hydrogels obtained from DEX with the same average molecular weight, but different oxyethylene chain length to highlight its effect on the swelling capacity. The increase in the molecular weight of PEG involves a change in the swelling properties. It is reasonable to assume that the derivative with PEG_500_MA as the lateral chain has greater ability to form clusters of oxyethylene chains with respect to thePEG_360_MA derivatives [24,25]. A greater number of oxyethylene units corresponds to a decreased rate of water entry into the polymeric lattice due to the increase in the hydrogel hydrophobicity. Indeed, the hydrophilic–hydrophobic balance in the polymer structure influences cluster formation [26], but also water entrance into the network.

**Figure 9:**
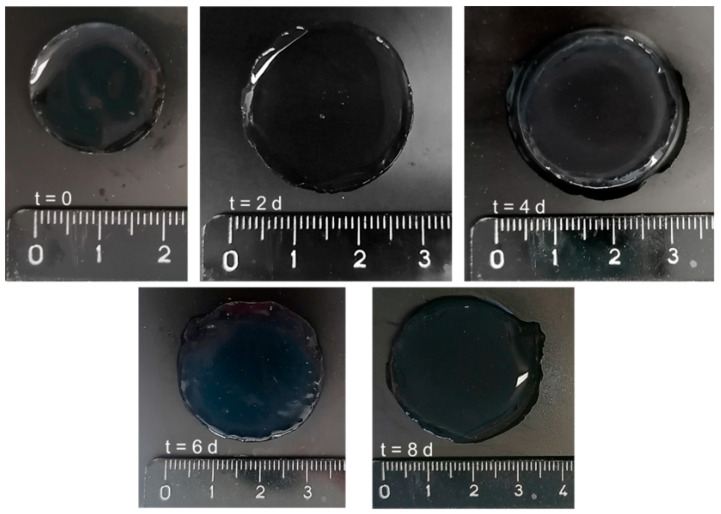
Pictures representing the swelling process of a DEX_40_-PEG_500_MA hydrogel in PBS (pH 7.4). The modification in the q value matches the variation in the dimensions of the hydrogel. Pictures were taken at time zero and after 2, 4, 6 and 8 days.

The different swelling ability shown by the hydrogels obtained from DEX_500_-PEG_360_MA with respect to DEX_40_-PEG_360_MA probably depends on the different degree of polymer chain entanglement, in agreement with the viscosity data of the corresponding starting solutions. By following the variation of the q value over time, it is possible to define the degradation profile of these hydrogel systems, which showed a progressive weight loss as a result of the consequent hydrolysis of the carbonate ester bonds and decay of the structure. All the samples decomposed completely within a few days, reaching the maximum value of q followed by a sharp decrease due to the complete degradation of the network [27]. This behavior depends on the gradual bulk erosion of the hydrogel matrix that is strictly related to the rate of water entrance. In particular, progressive bulk erosion was also confirmed by the images taken at different times during the swelling process in PBS. As an example, images of a DEX_40_-PEG_500_MA hydrogel at time zero and after two, four, six, and eight days in PBS are reported in Figure 9. It can be observed a continuous and progressive widening of the hydrogel, which matches and can justify the increase in the q value already discussed.

### 2.5. Release Studies

**Figure 10:**
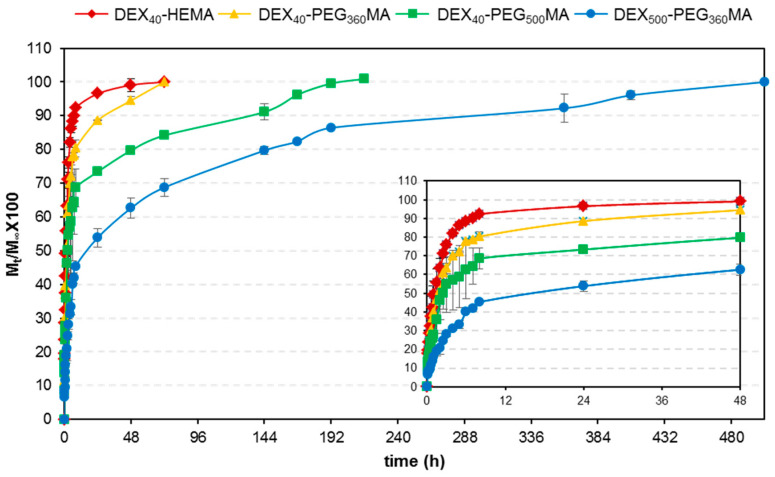
Release profiles of FITC-DEX from hydrogels fabricated with the different methacrylated DEX derivatives measured in PBS (pH 7.4) at 37.0 ± 0.5 °C. The insert represents an expanded region of the graph.

Release studies were carried out in PBS (pH 7.4) at 37.0 ± 0.5 °C on hydrogels loaded with a fluorescent dextran, employed as a large model molecule. The release profiles obtained from hydrogels prepared using different DEX derivatives are shown in Figure 10. A trend strictly linked to the swelling degree of the polymeric lattice and related to the rate of water entry into the hydrogel, is evident. The speed of water entrance is correlated to the hydrophobicity of the system and therefore it is possible to modulate the drug release simply by increasing the length of the oxyethylene chain.

The release profile from the hydrogels obtained from DEX at higher molecular weight is also reported in Figure 10. The effect of the molecular weight of DEX on the release profiles is much more evident. In particular, a modulation of the release over a period of 480 h is evident, and is also consistent with the obtained dynamic swelling data. In fact, even if the DEX_40_-PEG_360_MA and DEX_500_-PEG_360_MA gels reach the same swelling degree in terms of absolute q value, the time necessary to attain maximum swelling is deeply influenced by the molecular weight of the polymer. The entanglement of the polymer chains hinders the entrance of water, slowing down both the drug diffusion process and the matrix erosion.

Globally, both the molecular weight of DEX as well as the length of the linker are able to modify the degradation rate of these new DEX polymers and consequently the release time of a macromolecule loaded inside the corresponding hydrogels. Therefore, both these parameters can be modulated and adjusted to opportunely tailor the release of the incorporated drug and in this way achieve specific therapeutic needs. However, the DEX_500_ derivative possesses more adequate rheological properties to develop injectable and in situ crosslinkable drug delivery systems. Finally, the different polymeric derivatives can be opportunely blended and this strategy can be adopted to obtain the most suitable drug delivery systems, conveniently designed to meet specific therapeutic needs. Therefore, these new biodegradable methacrylated dextrans appear to possess interesting and promising properties for the development of effective delivery systems of large bioactive molecules. Additionally, further investigation is needed to evaluate the applicability of these polymers under physiological conditions and, in particular, their biological safety, which represents a fundamental issue of every material with potential application in the biomedical or pharmaceutical field. However, evidence exists on the biocompatibility of several different methacrylated DEX derivatives [18,28], which may also allow us to presume a good safety profile for these new biodegradable methacrylated dextrans.

## 3. Conclusions

A series of biodegradable DEX derivatives was synthesized coupling methacrylated oxyethylene chains to the polymeric backbone through a carbonate ester bond. The oxyethylene chain length was able to affect the rheological properties of the corresponding photo-crosslinked hydrogels, and was also able to modify the rate of water uptake, which is regulated by the balance between the hydrophilic and hydrophobic portions of the specific polymeric derivative. The longer the oxyethylene arm, the slower the swelling of the hydrogel. As a consequence of the slowing down of the entrance of water, the degradation rate decreases, and thus the release rate of a drug entrapped in the network. The rheological properties of the polymers as well as those of the corresponding hydrogels were also deeply affected by the molecular weight of the starting DEX. In particular, the viscosity of the polymer solution increased significantly when the molecular weight of the starting DEX was augmented. Anyway, the investigated polymer solution showed adequate injectability and, at the same time, was able to remain at the injection site without spreading. This rheological behavior is due to the higher degree of polymer chain entanglement, which is also responsible for the reduced rate of water uptake and, consequently, decreased degradation rate of the network. These characteristics are able to influence the release profile of a large drug molecule loaded into the degradable polymeric matrices. Globally, both the molecular weight of the polymer as well as the length of the lateral arm can influence the biodegradability of these new hydrogels, and can therefore be modulated and adjusted to achieve defined drug release kinetics. An opportune choice of the molecular weight of the starting polymer as well as of the number of oxyethylene units of the linker, or the use of mixtures of different DEX derivatives can represent interesting strategies to easily control how large molecules are released, and tailor them to meet specific therapeutic needs.

## 4. Materials and Methods

### 4.1. Materials

All used reagents were of analytical grade. Dextran (DEX) from *Leuconostoc* ssp. (Mr 40,000, DEX_40_ and 500,000, DEX_500_), hydroquinone mono-methyl ether, *N*,*N*’-carbonyldiimidazole (CDI), *N*-methyl-2-pyrrolidone, 4-dimethylaminopyridine (4-DMAP) were purchased from Fluka. Anhydrous dimethylsulfoxide (DMSO), DMSO-d6, anhydrous tetrahydrofuran (THF), ethanol (EtOH), 2-methoxyethanol, irgacure 2959, nicotinamide, fluorescein isothiocyanate-dextran (FITC-DEX, Mn 4000), hydroxyethyl methacrylate (HEMA), polyethylene glycol mono-methacrylate (Mn 360 and 500; PEG_360_-MA and PEG_500_-MA), D_2_O, and dialysis membranes (cut-off 12,000–14,000 Da) were purchased from Sigma-Aldrich (Saint Louis, MO, USA).

### 4.2. Synthesis of Dextran Derivatives

The synthesis was carried out in two steps, according to the procedure already reported in the literature for this type of functionalization [29]. HEMA (0.29 g; 2 mmol) was dissolved in anhydrous THF (7 mL) under a nitrogen atmosphere and subsequently CDI (0.37 g; 2 mmol) was added and left to react for 16 h at room temperature. Then, hydroquinone mono-methyl ether (0.25 g) was added to the mixture and the solvent was evaporated under reduced pressure, obtaining HEMA-IC (hydroxyethyl methacrylate N-imidazoylcarbamate), which was employed without purification. In the second step, DEX (2.4 g) was dissolved in anhydrous DMSO (18 mL), then mixed with 4-DMAP (0.47 g) and HEMA-IC. The mixture was kept reacting for 24 h under magnetic stirring at room temperature and then EtOH (200 mL) was added dropwise to precipitate the polymer. The suspension was filtered and the recovered solid was dissolved in water (15 mL). The solution, having a pH about 10, was neutralized with 0.1 M HCl and submitted to exhaustive dialysis against distilled water. The solution was frozen and then freeze-dried by employing a LIO 5P freeze-dryer (5 Pascal, Milan, Italy) equipped with a vacuum pump Adixen (Annecy, France). After freeze-drying, the polymer was characterized by FTIR and NMR. FTIR spectra were recorded with a Perkin Elmer Spectrum-One spectrometer (Waltham, MA, USA) in the range of 4000–650 cm^−1^. ^1^H- and ^13^C-NMR spectra were obtained with a Bruker Avance 400 spectrometer (Rheinstetten, Germany). Specifically, ^1^H-NMR spectra were obtained in a mixture D_2_O/DMSO-d6, while ^13^C-NMR investigation was carried out in DMSO-d6. The degree of derivatization (DD, number of methacrylic groups every 100 repetitive units of DEX) was calculated on the basis of the ^1^H-NMR spectrum using nicotinamide as the internal standard. The same synthetic procedure was employed for all the derivatives under study obtaining DEX_40_-HEMA, DEX_40_-PEG_360_MA, DEX_40_-PEG_500_MA, DEX_500_-PEG_360_MA, and DEX_500_-PEG_500_MA, respectively. The derivatives from DEX_500_ were precipitated from the reaction mixture using 2-methoxyethanol instead of ethanol. A DD% of 5 ± 1 was achieved for all polymeric derivatives.

### 4.3. Preparation of Hydrogels

The hydrogels were prepared through UV irradiation performed with a G.R.E. 125W Helios Italquartz (Milan, Italy), equipped with a mercury vapor lamp. Aqueous solutions of the different methacrylated polymers (10% *w*/*v*) were mixed with irgacure 2959 dissolved in *N*-methyl-2-pyrrolidone (1% *w*/*v*) and bubbled with nitrogen, then they were photo-crosslinked for 10 min.

### 4.4. Rheological Measurements

Rheological experiments were performed with a rheometer TA Discovery HR 1 (TA Instruments, New Castle, DE, USA). Viscosity curves of the polymeric solutions were obtained with a cone-plate geometry (diameter of 40 mm, α 1.005°, gap 27 µm) by applying shear stresses in the range of 0.01–1 Pa. Moreover, photochemical hydrogels (thickness of 0.4 cm) were submitted to oscillatory frequency sweep analysis using a serrated plate–plate geometry in the range of 0.01–1 Hz and working in the linear viscoelastic region assessed through preliminary strain sweep studies.

All hydrogels were also submitted to dynamic mechanical analysis (DMA) in compression. The sample was placed between the upper and lower tool of the plate–plate measuring system of the instrument and subjected to a uniaxial load, moving the upper plate at the constant speed of 10 µm/s.

All the experiments were carried out at least in triplicate at the temperature of 37.0 ± 0.1 °C.

### 4.5. Swelling and Degradability Studies

The swelling degree (q) of the hydrogels was determined in phosphate buffer solution (PBS, pH 7.4, ionic strength *I* = 0.1). Precisely weighed aliquots of each hydrogel were immersed in PBS at 37.0 ± 0.5 °C and left to swell. At defined time intervals, the liquid in excess was removed and the samples were weighed. The swelling degree was expressed as:

q = W_s_/W_d_ (1)

where W_s_ and W_d_ are the weights of the swollen and the dry hydrogel, respectively. The process of swelling was monitored up to the complete degradation of all gels. Each experiment was carried out in triplicate and the mean value was reported as ± the standard deviation.

### 4.6. Release Studies

Drug loaded hydrogels were obtained by adding fluorescent dextran (FITC-DEX, 0.2 mg/mL) to the polymeric solution (10% *w*/*v*) prior to irradiation. Hydrogels loaded with FITC-DEX were put in 10 mL PBS (pH 7.4) under magnetic stirring (100 rpm) at 37.0 ± 0.5 °C. At pre-established time points, a sample of the release medium (0.25 mL) was withdrawn and replaced with the same amount of fresh buffer. Each sample was diluted (1:10) in distilled water and the release of FITC-DEX was monitored with a Perkin Elmer FL 6500 fluorescence spectrophotometer (Waltham, MA, USA). The amount of model molecule was determined measuring the fluorescence emitted at 520 nm, after excitation at 490 nm. All the experiments were carried out in triplicate and the results were reported as mean values ± the standard deviation.

## Data Availability

The data presented in this study are available on request to the corresponding author.

## References

[1] Jabobs S., Nair A.B., Shah J., Sreeharsha N., Gupta S., Shinu P. (2021). Emerging role of hydrogels in drug delivery systems, tissue engineering and wound management. Pharmaceutics.

[2] Austin M.J., Rosales A.M. (2019). Tunable biomaterials from synthetic, sequence controlled polymers. Biomater. Sci..

[3] Li J., Mooney D.J. (2016). Designing hydrogels for controlled drug delivery. Nat. Rev. Mater..

[4] Naahidi S., Jafari M., Logan M., Wang Y., Yuan Y., Bae H., Dixon B., Chen P. (2017). Biocompatibility of hydrogel-based scaffolds for tissue engineering applications. Biotechnol. Adv..

[5] Li Q., Ning Z., Ren J., Liao W. (2018). Structural design and physicochemical foundations of hydrogels for biomedical applications. Curr. Med. Chem..

[6] Rehman W.U., Asim M., Hussain S., Khan S.A., Khan S.B. (2020). Hydrogel: A promising material in pharmaceutics. Curr. Pharm. Des..

[7] Sosnik A., Seremeta K.P. (2017). Polymeric hydrogels as technology platform for drug delivery applications. Gels.

[8] Raza F., Zafar H., Zhu Y., Ren Y., Ullah A., Ullah Khan A., He X., Han H., Aquib M., Boakye-Yiadom K.O. (2018). A review on recent advances in stabilizing peptides/proteins upon fabrication in hydrogels from biodegradable polymers. Pharmaceutics.

[9] Shoukat H., Buksh K., Noreen S., Pervaiz F., Maqbool I. (2021). Hydrogels as potential drug-delivery systems: Network design and applications. Ther. Deliv..

[10] Mallicka S.P., Sumana D.K., Singhb B.N., Srivastavab P., Siddiquia N., Yellaa V.R., Madhualc A., Vemuria P.K. (2020). Strategies toward development of biodegradable hydrogels for biomedical applications. Polym. Plast. Tech. Mat..

[11] Vermonden T., Censi R., Hennink W.E. (2012). Hydrogels for protein delivery. Chem. Rev..

[12] Bae K.H., Kurisawa M. (2016). Emerging hydrogel designs for controlled protein delivery. Biomater. Sci..

[13] Li Y., Yang H.Y., Lee D.S. (2021). Advances in biodegradable and injectable hydrogels for biomedical applications. J. Control. Release.

[14] Gao F., Jiao C., Yu B., Cong H., Shen Y. (2021). Preparation and biomedical application of injectable hydrogels. Mater. Chem. Front..

[15] Fan D.Y., Tian Y., Liu Z.J. (2019). Injectable hydrogels for localized cancer therapy. Front. Chem..

[16] Nguyen Q.V., Huynh D.P., Park J.H., Lee D.S. (2015). Injectable polymeric hydrogels for the delivery of therapeutic agents: A review. Eur. Polym. J..

[17] Bae K.H., Wang L.-S., Kurisawa M. (2013). Injectable biodegradable hydrogels: Progress and challenges. J. Mater. Chem. B.

[18] Van Tomme S.R., Hennink W.E. (2007). Biodegradable dextran hydrogels for protein delivery applications. Expert Rev. Med. Dev..

[19] Pacelli S., Paolicelli P., Casadei M.A. (2015). New biodegradable dextran-based hydrogels for protein delivery: Synthesis and characterization. Carbohydr. Polym..

[20] Van Dijk-Wolthuis W.N.E., Kettenes-van den Bosch J.J., van der Kerk-van Hoof A., Hennink W.E. (1997). Reaction of dextran with glycidyl methacrylate:  an unexpected transesterification. Macromolecules.

[21] Van Dijk-Wolthuis W.N.E., Franssen O., Talsma H., van Steenbergen M.J., Kettenes-van den Bosch J.J., Hennink W.E. (1995). Synthesis, characterization, and polymerization of glycidyl methacrylate derivatized dextran. Macromolecules.

[22] Lin-Gibson S., Bencherif S., Cooper J.A., Wetzel S.J., Antonucci J.M., Vogel B.M., Horkay F., Washburn N.R. (2004). Synthesis and characterization of PEG dimethacrylates and their hydrogels. Biomacromolecules.

[23] Pacelli S., Paolicelli P., Pepi F., Garzoli S., Polini A., Tita B., Vitalone A., Casadei M.A. (2014). Gellan gum and polyethyleneglycol dimethacrylate double network hydrogels with improved mechanical properties. J. Polym. Res..

[24] Azri A., Giamarchi P., Grohens Y., Olier R., Privat M. (2012). Polyethylene glycol aggregates in water formed through hydrophobic helical structures. J. Colloid Interf. Sci..

[25] Hammouda B., Ho D.L., Kline S. (2004). Insight into clustering in poly(ethylene oxide) solutions. Macromolecules.

[26] Chiang W.H., Lan Y.J., Huang Y.-C., Chen Y.W., Huang Y.F., Lin S.C., Chern C.S., Chiu H.C. (2012). Multi-scaled polymersomes from self-assembly of octadecanol-modified dextrans. Polymer.

[27] Van Dijk-Wolthuls W.N.E., Tsang S.K.Y., Kettenes-van den Bosch J.J., Hennink W.E. (1997). A new class of polymerizable dextrans with hydrolyzable groups: Hydroxyethyl methacrylated dextran with and without oligolactate spacer. Polymer.

[28] De Groot C.J., Van Luyn M.J., Van Dijk-Wolthuis W.N., Cadée J.A., Plantinga J.A., Den Otter W., Hennink W.E. (2001). In vitro biocompatibility of biodegradable dextran-based hydrogels tested with human fibroblasts. Biomaterials.

[29] De Jong S.J., De Smedt S.C., Demeester J., van Nostrum C.F., Kettenes-van den Bosch J.J., Hennink W.E. (2001). Biodegradable hydrogels based on stereocomplex formation between lactic acid oligomers grafted to dextran. J. Control. Release.

